# Systemic Disorders Closely Associated with Malocclusion in Late Adolescence: A Review and Perspective

**DOI:** 10.3390/ijerph19063401

**Published:** 2022-03-14

**Authors:** Masanobu Abe, Akihisa Mitani, Atsushi Yao, Kazuto Hoshi, Shintaro Yanagimoto

**Affiliations:** 1 Division for Health Service Promotion, The University of Tokyo, Tokyo 113-0033, Japan; mitania-int@h.u-tokyo.ac.jp (A.M.); yaoa-int@h.u-tokyo.ac.jp (A.Y.); yanagimoto@hc.u-tokyo.ac.jp (S.Y.); 2 Department of Oral & Maxillofacial Surgery, The University of Tokyo Hospital, Tokyo 113-8655, Japan; hoshi-ora@h.u-tokyo.ac.jp

Oral diseases such as dental caries and periodontal disease are reported to be associated with various systemic diseases such as heart disease, respiratory disease, diabetes, rheumatism, and metabolic syndrome, thus increasing the importance of prevention and early treatment [1,2,3,4,5]. On the other hand, the association between malocclusion and overall health has not been well documented. Recently, we screened subjects in late adolescence for systemic disorders associated with malocclusion [6,7]. We would like to summarize the literature, including our previous reports, to provide a new perspective on occlusion and overall health.

Malocclusion is defined as “deviation from normal occlusion”. However, the concept of malocclusion is broad and vague [8,9]. In our studies, we have classified malocclusion into two types: (I) occlusal disorder, which is a functional abnormality [6], and (II) teeth-alignment disorder, which is a morphological or cosmetic abnormality [7]. From the survey results of a questionnaire for freshmen enrolled at the University in Tokyo from 2017 to 2019, we extracted data from 9098 students under 20 years of age (17–19 years, mean age 18.3) and retrospectively examined the association of malocclusion with overall health histories. Among the 9098 students, 195 (2.1%) who had complaints of not being able to chew well were classified into the category of (I) occlusal disorder [6]. Among the 9098 students, 1915 (21.0%) students had complaints of having poorly aligned teeth. Of those 1915, there were 1819 who had no complaints of occlusal abnormality and were therefore classified into the category of (II) teeth-alignment disorder [7] (Figure 1).

The association of malocclusion with 18 systemic disorders, excluding acute illnesses and relatively rare diseases (e.g., those identified in fewer than 50 patients), were analyzed. The disorders were as follows: pollinosis, food/drug allergy, inhaled antigen allergy, allergic rhinitis, otitis media/externa, sinusitis, pneumothorax/mediastinal emphysema, asthma/cough-variant asthma, atopic dermatitis, urticaria, scoliosis, spondylosis/spondylolisthesis/hernia, strabismus, myopia/hyperopia/astigmatism, arrhythmia, abnormal electrocardiogram (ECG) other than arrhythmia, anemia, and gum bleeding.

As result of the analysis, histories of allergic rhinitis, asthma, and arrhythmia were found to be closely associated with (I) occlusal disorder, with high odds ratios of 2.18, 1.84, and 2.81, respectively, by multivariate analysis (logistic regression analysis) [6]. On the other hand, gum bleeding, which is a major symptom of periodontal disease, was found to be closely associated with (II) teeth-alignment disorder, with a high odds ratio of 1.54. In addition, pollinosis and gender (female) showed independent associations with teeth-alignment disorder, although the odds ratios were not so high (1.20 and 1.14, respectively) [7] (Figure 1).

Allergic rhinitis and pollinosis are both allergic diseases; allergic rhinitis was associated with occlusal disorders, while pollinosis was associated with teeth-alignment disorder. In both cases with allergic rhinitis and pollinosis, mouth breathing is considered a cause of malocclusion. Allergic rhinitis is a perennial disease and is considered to induce occlusal disorder via continuous mouth breathing [10,11]. On the other hand, pollinosis is a seasonal disease. Even when mouth breathing occurs, its duration is limited; thus, it is unlikely to cause occlusal disorder, even though teeth-alignment disorder may occur.

Close association between asthma and occlusal disorder has been demonstrated previously. In one study, malocclusion, particularly anterior open bite, was closely associated with asthma in adolescents [12]. Faria et al. analyzed the effects of asthma on dental and facial deformities and found that incompetent lip posture and an open nasal lip angle were significantly more frequent in patients with asthma than the controls. They also showed that patients with asthma had more dental crossbite, overbite, overjet, and smaller inter-bicuspid and inter-molar distances than the control group [13]. Although the mechanism underlying the association between asthma and malocclusion remains unclear, it is speculated that in the case with occlusal disorder, mouth breathing can induce asthma by direct stimulation of the airways by cold air or allergens. In asthma, unlike allergic rhinitis, mouth breathing is considered to be downstream of occlusal disorders [14,15].

Arrhythmia was closely associated with occlusal disorder. Recent studies have shown that chronic stress can be a risk factor for atrial fibrillation (AF), one of the most prevalent arrhythmias, through mechanisms such as increased inflammation and increased activity in the autonomic nervous system [16]. It has been suggested that occlusal disharmony due to missing teeth and other factors can lead to chronic stress and increase cardiac events [17,18,19]. Recently, Suita et al. investigated the relationship between occlusal disharmony (considered to be the same as occlusal disorder) and AF using bite-opening (BO) mice. AF susceptibility was increased concomitantly with atrial remodeling, including fibrosis, myocyte apoptosis, and oxidative DNA damage, in the BO mice [20,21]. This result suggests that cardiovascular diseases are deeply involved in malocclusion.

Gum bleeding, a typical symptom of periodontal disease, has been found to be associated with teeth-alignment disorder. Although there are several reports on the relationship between malocclusion and gingivitis in adolescence, the details are not clear [22,23]. Food residues and plaque resulting from difficulty in brushing due to irregularly positioned teeth may be involved in the induction of gingival inflammation [4]. In cases where there is no functional problem but the teeth are misaligned, correcting the teeth alignment may prevent or improve periodontal disease by improving toothbrush access and, hence, the cleanliness of teeth [24].

The association between occlusion and overall health has not received much attention in the past; however, in recent years, the importance of the association has begun to be demonstrated. Although further knowledge and clarification of the mechanisms involved are needed, improving malocclusion through orthodontic or prosthodontic treatment might contribute to the prevention and treatment of systemic diseases.

## Figures and Tables

**Figure 1:**
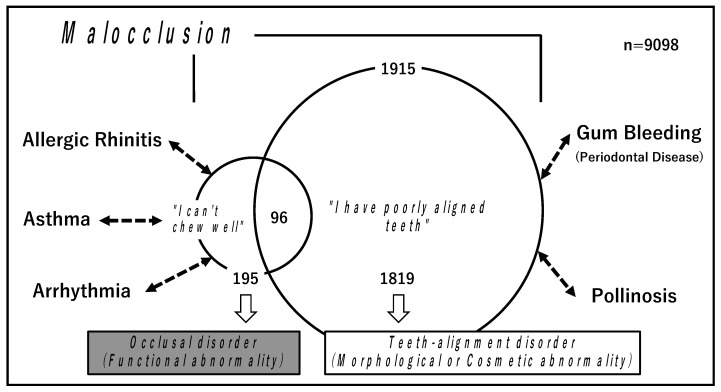
Malocclusion was classified as two types: (I) occlusal disorder, which is a functional abnormality, and (II) teeth-alignment disorder, which is a morphological or cosmetic abnormality. (I) Occlusal disorder was closely associated with histories of allergic rhinitis, asthma, and arrhythmia. On the other hand, (II) teeth-alignment disorder was closely associated with gum bleeding and pollinosis.
